# Treatment of Vulvovaginal Laxity by Electroporation: The Jett Plasma Medical for Her II Study

**DOI:** 10.3390/jcm12196234

**Published:** 2023-09-27

**Authors:** Tomas Fait, Tivadar Baltazár, Leona Bubenickova, Jan Kestranek, Martin Stepan, Miroslav Muller, Pavel Turcan

**Affiliations:** 1 Department of Obsterics and Gynaecology, 2nd Faculty of Medicine, Charles University, 150 00 Prague, Czech Republic; tfait@seznam.cz; 2 Department of Health Care Studies, College of Polytechnics Jihlava, 586 01 Jihlava, Czech Republic; 3 Department of Agrochemistry, Soil Science, Microbiology and Plant Nutrition, Faculty of AgriSciences, Mendel University in Brno, 613 00 Brno, Czech Republic; 4 Faculty of Medicine, Masaryk University, 625 00 Brno, Czech Republic; bubenickova@jett.eu; 5 Department of Obsterics and Gynaecology, Hospital Trutnov, 541 01 Trutnov, Czech Republic; kestranek.jan@nemtru.cz; 6 Department of Obsterics and Gynaecology, Medical Faculty in Hradec Kralove, Charles University, 500 05 Hradec Kralove, Czech Republic; martin.stepan@fnhk.cz; 7 Gynedology Pardubice Muller, 530 02 Pardubice, Czech Republic; info@gynmuller.cz; 8 Mediol Centre—Olomouc, 779 00 Olomouc, Czech Republic

**Keywords:** vaginal laxity, vulvovaginal laxity, nonsurgical vaginal tightening, incontinence, sexual function, urogenital distress, electroporation therapy

## Abstract

Introduction: Vaginal laxity is a widespread and undertreated medical condition associated especially with vaginal parity. Aim: To evaluate the efficacy and safety of electroporation therapy treatment of vulvovaginal laxity by the Jett Plasma for Her II device. Methods: The Jett Plasma for Her II Study is a multicentric, prospective, randomized, single-blinded, and controlled study. Women presenting with vaginal laxity were randomized to receive electroporation therapy delivered to the vaginal tissue (active—82 patients) vs. therapy with zero intensity (placebo—9 patients). Results: A total of 91 subjects whose average age was 48.69 ± 10.89 were included. Due to the results of a one-way analysis of variance, it may be concluded that in the case of the vaginal laxity questionnaire (VLQ), there is a statistically significant difference between actively treated patients and the placebo group (F_1,574_ = 46.91; *p* < 0.001). In the case of the female sexual function index (FSFI), a one-way ANOVA test also showed a statistically significant difference between the actively treated patients and the placebo group (F_1,278_ = 7.97; *p* = 0.005). In the case of the incontinence impact questionnaire-7 (IIQ-7), a one-way ANOVA test showed a statistically significant difference between the actively treated patients and the placebo group (F_1,384_ = 15.51; *p* < 0.001). It confirms that improvement of vaginal laxity is conjoined with benefits in symptoms of urinary incontinence. Biopsy performed after the end of the treatment shows an increase in the vaginal mucosa thickness by an average of 100.04% in the active group. The treatment was well tolerated with no adverse events. No topical anesthetics were required. Conclusions: Treatments of vulvovaginal laxity by electroporation therapy achieved significant and sustainable 12-month effectiveness. Responses to the questionnaires also suggest subjective improvement in self-reported sexual function, incontinence, sexual satisfaction, and urogenital distress.

## 1. Introduction

Vaginal laxity (vaginal looseness, vaginal relaxation syndrome, wide vagina syndrome) is a common condition characterized by a loss of tone or elasticity of the vagina [1]. This can be caused by a variety of factors, but the most important of them is vaginal childbirth [2]. It may also be partly caused by aging and oestrogen deficiency [3], but this idea is not generally approved. The pelvic floor muscles play a critical role in maintaining the vaginal tone and supporting the pelvic organs, but they can become weakened or damaged due to childbirth. As a result, the vaginal walls may become stretched or widened, leading to a decrease in excitement during sexual activity, difficulty achieving orgasm, and a loss of confidence [4,5]. 

Studies show prevalence of vaginal laxity is about 35%. Personal history of a single delivery relates to 5.6 (CI 1.67–15.3, *p* = 0.004) times more likely to report laxity than in nulliparous women. Women who had caesarean delivery were protected against vaginal laxity (adjusted odds ratio 0.39, 95% CI 0.17–0.9) [6]. There is an extraordinarily strong association with musculus levator ani extensive distensibility and all measures of anterior and posterior compartment descent [7]. However, it is not an early symptom of pelvic organ prolapse [8].

Aging is another common cause of vaginal laxity. As women age, their bodies undergo several changes, including a decrease in oestrogen levels. Oestrogen plays a crucial role in vaginal health and elasticity, and a decrease in oestrogen can lead to thinning of the vaginal walls and decreased lubrication. These changes can contribute to vaginal laxity and discomfort during sexual activity. On the other side, the main sign of vulvovaginal atrophy (genitourinary syndrome of menopause) caused by oestrogen deficiency is vaginal tightness [9].

In addition to childbirth and aging, obesity is another risk factor for vaginal laxity. Women who are overweight or obese may experience increased pressure on their pelvic floor muscles, leading to stretching and weakening. This can contribute to symptoms of vaginal laxity and may increase the risk of urinary incontinence and other pelvic floor disorders [10].

There are several treatment options available for vaginal laxity, ranging from non-invasive methods such as pelvic floor exercises and vaginal rejuvenation devices to more invasive approaches like vaginal surgery [11]. Pelvic floor physical therapy is a common nonsurgical treatment option that involves exercises designed to strengthen the pelvic floor muscles and improve vaginal tone. 

Vaginal rejuvenation devices, such as thermal therapy and radiofrequency energy, can also be used to stimulate collagen production and tighten the vaginal walls [12]. 

Jet Plasma for Her II (Compex Ltd., Brno, Czech Republic) works on the basis of electroporation. The base of this mechanism is the creation of small temporary nanopores in the cell walls. It is induced by the application of high voltage electrical pulse to the cell membrane. Macromolecules and other ions could pass through these nanopores in both directions. The cells increase their volume [13]. It decreases the atrophy of vaginal mucosa and submucosal tissue and improves the tissue cohesion. Electroporation is also used for the transport of drugs into tumor cells or in irreversible ones for the destruction of cells. In this situation, we used another voltage and length of application [14]. 

The purpose of the Jett Plasma for Her II study was to determine the efficacy and safety of this type of therapy in women with vaginal laxity. We have three years of experience with this type of therapy. We started with this therapy in 2020 when a vaginal application device was created. The same therapy has been used in beauty care at Jett Plasma Lift Medical since 2015 with other devices.

## 2. Materials and Methods 

The Jett Plasma for Her II Study is a multicentric, prospective, randomized, single-blinded, and controlled study which started in November 2019 and currently includes 91 patients. The study has been authorized by the Ethics Committee of University Hospital Ostrava, code number 16.10-TF-JPH II. The study was completed without any additional payment from patients or reimbursement for patients.

Healthy adult female subjects who clinically present with vaginal laxity (it was only one inclusion criterion) and expressed interest in treatment were considered eligible for the study. The patients were offered participation in the study by their attending physicians. 

Inclusion criteria for subject selection consisted of voluntarily signed informed consent, age at least 18 years, a negative pregnancy test, self-reported perceptions of vaginal laxity defined on the vaginal laxity questionnaire (VLQ), and Papanicolaou smear cytology showing no dysplasia within 36 months prior to the treatment. 

Women with evidence of epilepsy, pregnancy, metal implants in the treated area, skin diseases or inflammations in the treatment area, urinary tract infection, collagen vascular disease, oncological disease in the vulvovaginal region, any untreated/badly treated disease in vulvovaginal region, birth defects of vagina, stenosis and strictures of the vagina, synechia of vulva, previous reconstructive vaginal surgery, vaginal lasers, or vaginal injections of fat or fillers within 6 months, BMI ≥ 35 were excluded. 

Prior to the treatment and at the 1, 3, 6, and 12 month follow-up visits, participants completed a packet of self-report questionnaires to characterize and follow the effects of treatment. Five validated questionnaires were included in the packet: the vaginal laxity questionnaire (VLQ), the female sexual function index (FSFI), the sexual satisfaction questionnaire (SSQ), and short forms of the urogenital distress inventory (UDI-6) and the incontinence impact questionnaire (IIQ-7). 

In 25 patients out of the total number of patients, vaginal mucosa biopsies were performed. The first biopsy took place before the treatment, and the second sample was taken 3 months after the third treatment. 

Treatments were performed three times by the vaginal probe of Jett Plasma for Her II (Compex, Ltd., Brno, Czech Republic) with a time interval of 10 to 14 days. It uses an electric current of 2.8 mA with a voltage of 5 kV. It induces a maximum temperature of 45 °C in the tissue of the vagina wall. Each application takes approximately 7.5 min. 

### 2.1. Questionnaires

The vaginal laxity questionnaire has seven-level ordered responses (1—very loose, 2—moderately loose, 3—slightly loose, 4—neither loose nor tight, 5—slightly tight, 6—moderately tight, or 7—very tight). For the primary efficacy endpoint, “no vaginal laxity” was classified as a VLQ score of at least 5 (i.e., ≥5) [12].

The incontinence impact questionnaire (IIQ-7) assesses the psychosocial impact of incontinence in women. It consists of 7 items: 1—household chores, 2—physical recreation, 3—entertainment activities, 4—travel > 30 min away from home, 5—social activities, 6—emotional health (nervousness, depression, etc.), and 7—feeling frustrated, which are subdivided into 4 domains: PA—physical activity (items 1 and 2), TR—travel (items 3 and 4), SA—social activities (item 5), and EH—emotional health (items 6 and 7). The total score is in the range of 0–100 [15].

The urogenital distress inventory (UDI-6) is a condensed version of a condition-specific quality of life instrument, UDI. Presently, UDI-6 is much more often used than its longer version. UDI-6 consists of 6 items: 1—frequent urination, 2—leakage related to the feeling of urgency, 3—leakage related to activity, coughing, or sneezing, 4—lesser amounts of leakage (drops), 5—difficulty emptying the bladder, and 6—pain or discomfort in the lower abdominal or genital area. Higher scores in UDI-6 indicate higher disability. The total score is from 0 to 100 [15]. 

The female sexual function index (FSFI) is a validated instrument for the assessment of sexual function, consisting of 19 questions. The questions are grouped for domains of libido, arousal, lubrication, orgasm, satisfaction, and pain; higher scores reflect better sexual function (maximum score 36). An FSFI total score less than or equal to 26.5 is recognized in the medical literature as indicating female sexual dysfunction (FSD) [16].

The sexual satisfaction questionnaire (SSQ) assesses sexual satisfaction from vaginal intercourse. SSQ has six-level ordered responses (1—excellent sexual satisfaction, 2—very good sexual satisfaction, 3—good sexual satisfaction, 4—fair sexual satisfaction, 5—poor sexual satisfaction, and 6—no sexual satisfaction) [17]. 

Patients evaluated the pain of the procedure by a visual analogue scale of 0, painless, to 10, extremely painful [18]. 

### 2.2. Randomization

The assignment to the placebo or the active group was generated using the online tool https://www.random.org/lists/ (accessed on 1 November 2019), where numbers from 1 to 140 were randomly assigned to either the active treatment (90 occurrences) or the placebo treatment (50 occurrences). Physicians obtained a randomization letter for 10 patients. Each physician assigned a treatment to the patient according to the date of study entry, meaning that the first patient who entered the study was numbered 1, etc.

If a patient agreed to the biopsy, she was assigned a number 141–150, and all those patients underwent active treatment.

At the end of the study, each patient completed a blinding questionnaire to assess the blinding.

### 2.3. Statistical Analysis

All statistical analyses were performed using freely available software R, version 4.3.1. (https://mirrors.nic.cz/R/, CZ.NIC, Prague, Czech Republic) [19]. Package “ggplot2” [20] was used for creating advanced statistical graphs. To statistically evaluate several questionnaires (VLQ, FSFI, IIQ-7, UDI-6, and SSQ) with dependence on actively treated patients and the placebo group (or treatment time), a one-way analysis of variance (ANOVA) type I (sequential) sum of squares at a significance level of 0.05 was used [21]. 

To detect the difference among factor level means, Tukey’s honestly significant difference (HSD) test and “treatment contrasts” for calculating the factor level means with 95% confidence intervals CI was used [22]. Pearson’s chi-squared test of independence was used to check whether the observed frequency of vaginal laxity or sexual dysfunction occurrence (no or yes) differs significantly from the expected frequencies with dependence of method (actively treated patients and the placebo group). In the case of FSFI, a one-way analysis of covariance (ANCOVA) was performed to characterize the relationship between the age of the patient, treatment time, and type of method (actively treated patients and the placebo group). A coefficient of determination (R^2^) was used to explain how well one can predict the variation in the values of FSFI questionnaire score using the age of patients [22]. After the analysis, the assumptions of all our statistical models were also checked at a significance level of 0.05 with the help of different statistical tests and several diagnostic plots [23].

## 3. Results

A total of 91 patients (aged 26 to 77, median 48.6 years) were involved in this trial with average age of 48.6 (SD = 10.8) years. The average age in the group of actively treated patients was 47.7 (95% CI: 45.4, 50.1; *n* = 82) and 56.5 (95% CI: 49.6, 63.5; *n* = 9) in the case of the placebo group. This difference (F_1,87_ = 5.68; *p* = 0.02) was statistically significant only at 5% significance level.

**Figure 1:**
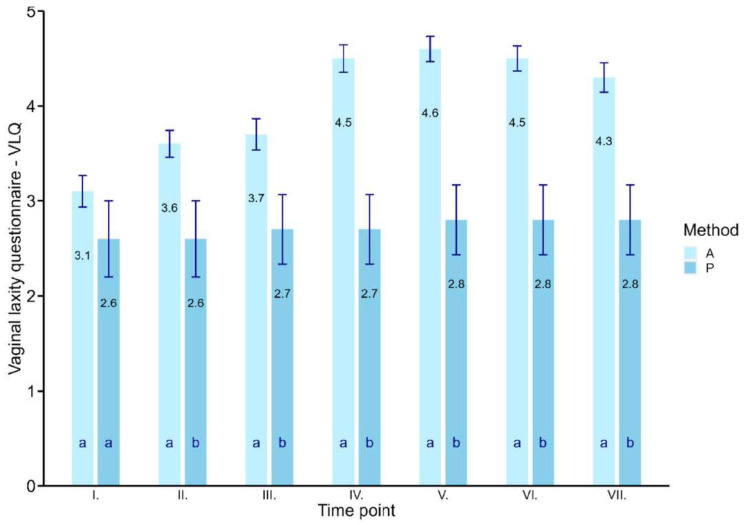
Mean values with standard error of the mean (SEM) for the vaginal laxity questionnaire VLQ with the dependence of “method” and “time point” (I.—1st treatment, II.—2nd treatment, III.—3rd treatment, IV.—1st control, V.—2nd control, VI.—3rd control, VII.—4th control). A—active, P—placebo. (Letters show statistically significant difference between actively treated patients and placebo group at significance level of 0.05).

Due to the results of the one-way analysis of variance, it can be concluded that in the case of the vaginal laxity questionnaire (VLQ), there is a statistically significant difference between the actively treated patients and the placebo group (F_1,574_ = 46.91; *p* < 0.001). The average value of the VLQ in the case of actively treated patients was 3.98 (95% CI: 3.86, 4.11) and in the case of the placebo group, 2.68 (95% CI: 2.33, 3.03), which is 32.6% less. The average improvement between the first treatment and the last follow-up was 1.22 points from 3.15 (SD = 10.8) to 4.27 (SD = 1.37). The 28.6% increase is statistically significant (F_6,506_ = 12.37; *p* < 0.001). However, the largest difference was found between the first treatment and the second control (diff: −1.43, *p* < 0.001). The highest VLQ value was observed in the case of the second control with an average of 4.57 (95% CI: 4.25, 4.89). The placebo group improved by 0.22 points from 2.56 (SD = 1.24) to 2.78 (SD = 1.09), i.e., 4%, which is not statistically significant (F_6,56_ = 0.09; *p* = 1). The differences between the active treatment and the placebo group were also statistically significant (except for the first treatment) in all follow-up controls (*p* < 0.05). The primary efficacy analysis of the VLQ is presented in Table 1 and Figure 1. The percentage of subjects in the actively treated patients reporting no vaginal laxity (VLQ score ≥ 5) at the first treatment was 15.6% (13 of 82) compared with 0% (0 of 9) in the placebo group, which should not be statistically different (χ^2^ _(1, *n*=89)_ = 1.41, *p* = 0.24). The largest percentage was achieved in the case of the second control, where actively treated patients reported no vaginal laxity (VLQ score ≥ 5) with a percentage of 45.7% (32 of 70) compared with 0% (0 of 9) in the placebo group. This difference showed borderline significance (χ^2^ _(1, *n*=79)_ = 3.97, *p* = 0.05).

In the case of the female sexual function index (FSFI), the one-way ANOVA also showed a significant difference between actively treated patients and the placebo group (F_1,278_ = 7.97; *p* = 0.005). The average value of FSFI in actively treated patients was 28.6 (95% CI: 27.9, 29.2) and in the placebo group, 26.0 (95% CI: 24.3, 27.7), which is 2.6% less. The average improvement between the first treatment and the last follow-up was 3.81 points from 25.6 (SD = 6.66) to 29.4 (SD = 5.4). The 12.9% increase is significant (F_4,235_ = 6.68; *p* < 0.001). However, the largest difference was found between the first treatment and the third control (diff: −4.32, *p* < 0.001). The largest FSFI value was observed in the case of the third control on average, with 29.9 (95% CI: 28.4, 31.4). The placebo group improved by 1.4 points from 25.1 (SD = 4.81) to 26.5 (SD = 5.39), i.e., 5.3%, which is not statistically significant (F_4,35_ = 0.11; *p* = 0.98). The differences between the active treatment and the placebo group were only statistically significant in the case of the first and second controls (*p* < 0.05). The primary efficacy analysis of FSFI is presented in Table 2. The percentage of subjects in the actively treated patients reporting no sexual dysfunction (FSFI score ≥ 26.5) at the first treatment was 46.6% (27 of 58) compared with 37.5% (3 of 8) in the placebo group, which is not different (χ^2^ _(1, *n* = 66)_ = 0.09, *p* = 0.76). The largest percentage was achieved in the case of the third control, where 84.1% (37 of 44) of the actively treated patients reported no sexual dysfunction (FSFI score ≥ 26.5) compared to 50% (4 of 8) in the placebo group. This difference is not statistically significant (χ2 _(1, *n* = 52)_ = 0.64, *p* = 0.42) either.

The IIQ-7 and UDI-6 questionnaires confirm that improvement of vaginal laxity is conjoined with benefits in symptoms of urinary incontinence. In the case of the incontinence impact questionnaire-7 (IIQ-7), a one-way ANOVA also showed a significant difference between the actively treated patients and the placebo group (F_1,384_ = 15.51; *p* < 0.001). The average value of IIQ-7 in actively treated patients was 12.0 (95% CI: 9.94, 14.06) and in the placebo group, 24.1 (95% CI: 18.42, 29.77), which is 50% higher. The average change between the first treatment and the last follow-up was 20.64 points from 26.05 (SD = 22.03) to 5.41 (SD = 14.08). Such a 79.2% decrease is statistically significant (F_4,336_ = 17.0; *p* < 0.001). However, the largest difference was registered between the first treatment and the first control (diff: −16.92, *p* < 0.001). The largest IIQ-7 value was observed in the case of the first treatment on an average of 26.05 (95% CI: 22.19, 29.91). The placebo group improved by 2.62 points from 26.19 (SD = 22.87) to 23.57 (SD = 24.04); this 10% decrease is not significant (F_4,40_ = 0.02; *p* = 0.99). The differences between the active treatment and the placebo group were statistically significant in all follow-up controls (*p* < 0.05). 

Due to the result of the one-way ANOVA, it can be concluded that in the case of the urogenital distress inventory (UDI-6), there is a statistically significant difference between the actively treated patients and the placebo group (F_1,385_ = 9.22; *p* = 0.003). The average value of UDI-6 in the case of an actively treated patient was 13.5 (95% CI: 11.91, 15.07) and in the case of the placebo, 20.7 (95% CI: 16.30, 25.01), which is 34.8% higher. The average change between the first treatment and the last follow-up was 21.3 points from 27.8 (SD = 17.1) to 6.52 (SD = 9.8). This 76.6% decrease is statistically significant (F_4,337_ = 33.15; *p* < 0.001). However, the largest difference was found between the first treatment and the first control (diff: −16.21, *p* < 0.001). The largest UDI-6 value was observed in the case of the first treatment average of 27.8 (95% CI: 24.94, 30.62). The placebo group improved by 2.47 points from 22.61 (SD = 12.43) to 20.17 (SD = 14.55); this 11% decrease is not statistically significant (F_4,40_ = 0.05; *p* = 0.99). The differences between the active treatment and the placebo group were statistically significant (except for the first treatment) in all follow-up controls (*p* < 0.05). 

**Figure 2:**
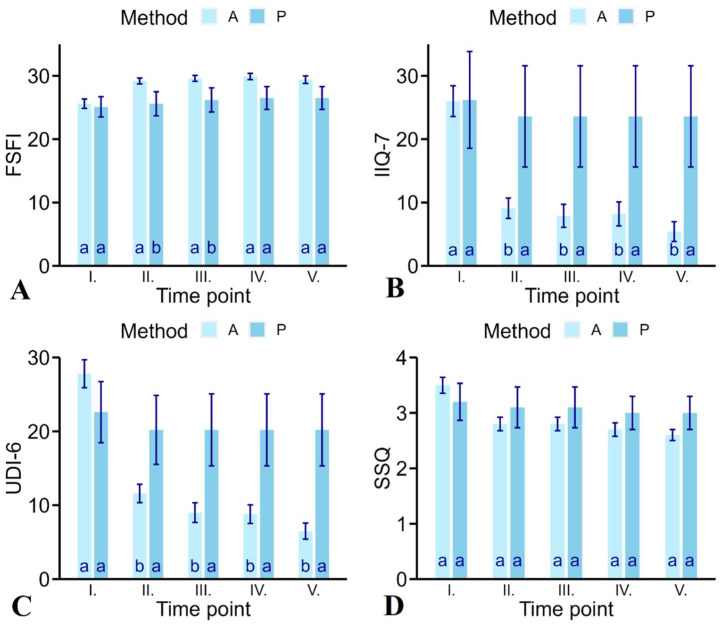
Mean values with standard error of the mean (SEM) for female sexual function index FSFI (**A**), incontinence impact questionnaire-7 IIQ-7 (**B**), urogenital distress inventory UDI-6 (**C**), and sexual satisfaction questionnaire SSQ (**D**) with the dependence of “method” and “time point” (I. –1st treatment, II.—1st control, III.—2nd control, IV.—3rd control, V.—4th control). A—active, P—placebo. (Letters show statistically significant difference between actively treated patients and placebo group at significance level of 0.05).

Different results were observed in the case of the sexual satisfaction questionnaire (SSQ) because, in this case, a one-way ANOVA did not detect a statistically significant difference between the actively treated patients and the placebo group (F_1,315_ = 0.80; *p* = 0.37). The average value of SSQ in the case of an actively treated patient was 2.93 (95% CI: 2.79, 3.10) and in the case of the placebo group, 3.10 (95% CI: 2.75, 3.45), which is 5% higher compared to the actively treated patients. The average change between the first treatment and the last follow-up was 0.92 points from 3.54 (SD = 1.28) to 2.62 (SD = 0.9). This 26% decrease is statistically significant (F_4,272_ = 6.78; *p* < 0.001). However, the largest difference was found between the first treatment and the fourth control (diff: 0.91, *p* < 0.001). The largest SSQ value was observed in the case of the first treatment average of 3.54 (95% CI: 3.27, 3.81). The placebo group improved by 0.25 points from 3.25 (SD = 1.04) to 3 (SD = 0.93); this 7.7% decrease is not statistically significant (F_4,35_ = 0.08; *p* = 0.99). The differences between the active treatment and the placebo group were not statistically significant either in all follow-up controls (*p* > 0.05) (Figure 2).

**Figure 3:**
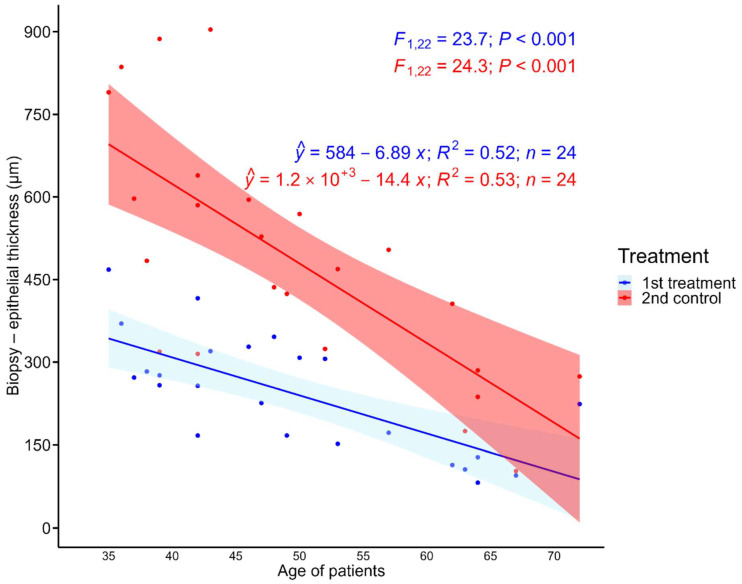
Negative linear relationship between the biopsy: epithelial thickness (μm) and the age of patients with dependence on “Treatment”.

A one-way ANOVA showed a statistically significant difference in vaginal mucosa thickness in samples before and after treatment (F_1,48_ = 26.57; *p* < 0.001). The average value of vaginal mucosa thickness in the first treatment was 241.4 (95% CI: 173.6, 309.1), and in the second control, 486.8 (95% CI: 419.1, 554.6), which is 50% higher compared to the first treatment. Results of the analysis of covariance also detected not only the impact of the treatment duration (F_1,45_ = 44.1; *p* < 0.001) but also the role of age (F_1,45_ = 39.18; *p* < 0.001). This relationship is medium-strong, assessed by the coefficient of determination (r^2^ = 52) in both treatments. A negative correlation was also found between age and vaginal mucosa thickness; the older the patient, the lower the value of vaginal mucosa thickness (Figure 3). Biopsy performed after the end of the treatment showed an increase in the vaginal mucosa thickness by an average of 112.6% (SD = 75.4) in the case of actively treated patients.

On average, a total of 80.6% felt no pain during the procedure. Only one patient rated the treatment as 5. In the case of the actively treated patients, this ratio was 70.1%. In the case of the first treatment, painlessness was 76.9% (74.3% in actively treated patients); in the case of the second treatment, it was 79.1% (76.8% in actively treated patients), and in the case of the third treatment it was 85.7% (84.1% in actively treated patients). The differences between the actively treated patients and the placebo group were significant only at a 5% significance level in all cases: first treatment (χ^2^ _(1, *n* = 91)_ = 6.41, *p* = 0.01), second treatment (χ^2^ _(1, *n* = 91)_ = 6.62, *p* = 0.01), and third treatment (χ^2^ _(1, *n* = 91)_ = 7.23, *p* = 0.01). In the first treatment, the pain was 0.33 (95% CI: 0.19, 0.48; *n* = 91) on a visual analogue scale, 0.26 (95% CI: 0.12, 0.41; *n* = 91) in the second treatment, and 0.16 (95% CI: 0.01, 0.31; *n* = 91) in the third treatment. There was no statistically significant difference among the treatments (F_2,257_ = 1.30; *p* = 0.27).

After the fourth follow-up control, 90% of women were satisfied with the treatment, and the average value of the satisfaction scale was 4.5 (SD = 0.68). 

## 4. Discussion

The main advantage of our study is that it is the first study using electroporation for the treatment of vaginal laxity. Additionally, the use of a control (placebo) subgroup is not common in other vaginal laxity treatment studies. On the other side, the size of the control group and the 26.3% drop-out of patients not during the therapy but during the follow-up may be considered weaknesses. The weaknesses of the study are also unknown hormonal status and sexual partnership. Another weakness could be missing data such as parity status and birth weight of patients’ children, but we believe that it is important for the cause of vaginal laxity, not for its therapy. 

Vaginal laxity is a topic that has generated significant attention in recent years, with many women expressing concerns about the perceived looseness of their vaginal muscles. While some people may view this as a purely cosmetic concern, it can have a real impact on a woman’s sexual health and overall well-being. 

The symptoms of vaginal laxity can include decreased sexual sensation, reduced sexual satisfaction, difficulty achieving orgasm, and urinary incontinence. While these symptoms can have a significant impact on a woman’s quality of life, they can often be effectively treated using a variety of medical interventions. 

To treat a wide vagina sensation, perineoplasty can be successfully used with low complication rates. Patients report high satisfaction and anatomical repair [24,25]. Perineoplasty is surgery removing excess or ruined skin and mucosa of the vaginal entrance. The repairing of supporting muscles is conjoined. It takes approximately 30 to 45 min in local or general anaesthesia. In a retrospective study of 38 women with a 6-month follow-up, the success rate of the perineoplasty procedure was 87.9%; according to a visual analogue scale, the partner satisfaction rate was 92.6%. Ten percent of patients said they had experienced dyspareunia at the introitus of the vagina during sexual intercourse [26].

There is no scientific evidence to prove the efficacy of over-the-counter vaginal tightening products, injectable volumizers, and physical devices such as silicon threads [27,28,29]. Pelvic physiotherapy is an accepted intervention for pelvic organ prolapse as well as vaginal laxity in the form of pelvic floor muscle training (PFMT) and Kegel exercises [30,31]. 

Energy-based devices (EBD) attempt to induce favorable changes in tissue using heat in the range of 40–42 °C. These devices restore the elasticity of connective tissue of the vaginal wall. At the same time, they improve vaginal lubrication and humidity of the vaginal mucosa. The procedures of energy-based vaginal rejuvenation are non-invasive and take from 8 to 30 min. The procedure is painless. Two or three sittings are recommended, spaced a month apart. A touch-up sitting is usually performed after 12–18 months. Indication for these procedures is vaginal laxity, vaginal dryness, mild symptoms of urinary incontinence, overactive bladder, low-grade prolapse, and orgasmic dysfunction [32]. 

Energy-based therapy could be based on LASER or radiofrequency. In a recent review, 59 studies were evaluated with 3609 women [33]. Minimally ablative fractional laser therapy was recognized as a safe, accurate, and efficient approach for resurfacing and regeneration of the skin. The most widely used lasers in vaginal tissues are the CO_2_ (10,600 nm) and the erbium: yttrium-aluminium-garnet (Er: YAG) laser. In 2013, an EBD was validated by the North American Menopause Society. The North American Menopause Society acknowledged the use of lasers for therapy of the genitourinary syndrome of menopause in 2013. However, results are frequently obtained from small, short-term studies without randomization [34,35].

For example, Gao et al. published a descriptive study without controls. A total of 29 patients were enrolled and treated with two sessions of FemTouch vaginal fractional CO_2_ laser, with a one-month interval between the sessions. Both subjective and objective measurements, including female sexual function index (FSFI), vaginal health index score (VHIS), vaginal tactile imaging (VTI), and histology, were used to validate the clinical efficacy and biophysical benefits after treatment. Results: The overall FSFI scores and VHIS scores after the first and second treatment sessions were significantly higher than the baseline scores (*p* < 0.01, *n* = 29). VTI measurements showed a significant increase in maximal pressure resistance (kPa) of both the anterior and posterior vaginal walls at a 10–12-month post-treatment visit compared with pre-treatment controls (*p* < 0.001; *n* = 16). Histological examination showed that laser treatment led to increases in the thickness of the stratified squamous epithelium layer and the density of connective tissues in the lamina propria [36].

Monopolar radiofrequency (RF) treatment with cryogenic surface cooling offers another less-invasive ambulatory therapy for vaginal laxity. The density of small nerve fibers in the papillary dermis increased after the application of RF. The biopsy test has shown neocollagenesis and neoelastogenesis in the submucosa and the development of new elastin. The Vaginal Introitus’s Viveve Treatment to Evaluate Effectiveness (VIVEVE I) trial was the first randomized control trial with radiofrequency in the therapy of vaginal laxity. A single RF treatment was proven to be safe and was associated with the improvement of vaginal laxity and sexual function [37].

The treatment relies on the concept that carefully controlled heat energy can be used to reach deeper submucosal tissue. The therapeutic goal is to stimulate connective tissue activation with subsequent tissue revitalization. It is the same situation as for the skin in beauty treatment. A similar process of neoelastogenesis and neocollagenesis might also occur in vaginal tissue. 

Some studies used a sheep vagina as an animal model. RF treatment procedures identical to those used in this human report were evaluated in serial tissue biopsies. Stromal remodeling with fibroblast activation in soft tissue was identified between one week and one month after the treatment. The increased submucosal and/or muscularis collagen was focally present over 6 months after treatment. The absence of ulceration, regional necrosis, and effacing dense collagen scarring over the 6-month follow-up period supports an acceptable safety profile for this treatment regimen [38].

## 5. Conclusions

This study confirms that a nonsurgical, nonablative treatment with Jett Plasma Medical for Her II, a new type of energy-based device working with electroporation, is a well-tolerated and safe procedure shown to produce statistically significant and clinically important improvements in vaginal laxity, incontinence, and improved sexual satisfaction in women in the active group compared to placebo treatment. This effect was prolonged until 12 months after the treatment. 

## Figures and Tables

**Table 1:** Results of analysis of contingence table with the help of Pearson’s Chi-squared test of independence in the case of the vaginal laxity questionnaire (VLQ). A—active, P—placebo.

	Method	Total	No Laxity	No Laxity (%)	Chisq Test	*p*-Value
1st treatment	A	82	13	15.9	1.41	0.24
	P	9	0	-		
2nd treatment	A	81	14	17.3	1.53	0.22
	P	9	0	-		
3rd treatment	A	80	24	30	2.64	0.10
	P	9	0	-		
1st control	A	78	34	43.6	3.80	0.05
	P	9	0	-		
2nd control	A	70	32	45.7	3.97	0.05
	P	9	0	-		
3rd control	A	66	27	40.9	3.55	0.06
	P	9	0	-		
4th control	A	56	22	39.3	3.40	0.07
	P	9	0	-		

**Table 2:** Results of the analysis of the contingence table with the help of Pearson’s Chi-squared test of independence in the case of the female sexual function index (FSFI). A—active, P—placebo.

Treatment	Method	Total	No Dysfunction	No Dysfunction (%)	Chisq Test	*p*-Value
1st treatment	A	58	27	46.6	0.09	0.76
	P	8	3	37.5		
1st control	A	56	42	75.0	0.99	0.32
	P	8	3	37.5		
2nd control	A	48	38	79.2	0.51	0.48
	P	8	4	50.0		
3rd control	A	44	37	84.1	0.64	0.42
	P	8	4	50.0		
4th control	A	34	26	76.8	0.41	0.52
	P	8	4	50.0		

## Data Availability

Data is unavailable due to ethical restrictions. Anonymized data are accessible on demand on authors mail address.
